# Permeation Increases Biofilm Development in Nanofiltration Membranes Operated with Varying Feed Water Phosphorous Concentrations

**DOI:** 10.3390/membranes12030335

**Published:** 2022-03-18

**Authors:** Luisa Javier, Laura Pulido-Beltran, Johannes S. Vrouwenvelder, Nadia M. Farhat

**Affiliations:** 1 Water Desalination and Reuse Center (WDRC), Division of Biological and Environmental Science and Engineering (BESE), King Abdullah University of Science and Technology (KAUST), Thuwal 23955-6900, Saudi Arabia; luisa.javier@kaust.edu.sa (L.J.); laura.pulidobeltran@kaust.edu.sa (L.P.-B.); johannes.vrouwenvelder@kaust.edu.sa (J.S.V.); 2 Department of Biotechnology, Faculty of Applied Sciences, Delft University of Technology, Van der Maasweg 9, 2629 HZ Delft, The Netherlands

**Keywords:** phosphate limitation, phosphorus quantification, biofouling, biofilm development, membrane filtration

## Abstract

Nutrient limitation has been proposed as a biofouling control strategy for membrane systems. However, the impact of permeation on biofilm development under phosphorus-limited and enriched conditions is poorly understood. This study analyzed biofilm development in membrane fouling simulators (MFSs) with and without permeation supplied with water varying dosed phosphorus concentrations (0 and 25 μg P·L^−1^). The MFSs operated under permeation conditions were run at a constant flux of 15.6 L·m^2^·h^−1^ for 4.7 days. Feed channel pressure drop, transmembrane pressure, and flux were used as performance indicators. Optical coherence tomography (OCT) images and biomass quantification were used to analyze the developed biofilms. The total phosphorus concentration that accumulated on the membrane and spacer was quantified by using microwave digestion and inductively coupled plasma atomic emission spectroscopy (ICP-OES). Results show that permeation impacts biofilm development depending on nutrient condition with a stronger impact at low P concentration (pressure drop increase: 282%; flux decline: 11%) compared to a higher P condition (pressure drop increase: 206%; flux decline: 2%). The biofilm that developed at 0 μg P·L^−1^ under permeation conditions resulted in a higher performance decline due to biofilm localization and spread in the MFS. A thicker biofilm developed on the membrane for biofilms grown at 0 μg P·L^−1^ under permeation conditions, causing a stronger effect on flux decline (11%) compared to non-permeation conditions (5%). The difference in the biofilm thickness on the membrane was attributed to a higher phosphorus concentration in the membrane biofilm under permeation conditions. Permeation has an impact on biofilm development and, therefore, should not be excluded in biofouling studies.

## 1. Introduction

It has been over 60 years since the first significant membrane application when a German manufacturer developed a microfiltration membrane for industrial purposes. Years later, in the late 1950s, a fundamental breakthrough in membrane science came when Loeb and Sourirajan discovered reverse osmosis membranes for water desalination [1,2,3]. Since then, humanity has benefited from significant advancements in membrane technologies. One advancement to address clean water scarcity is the use of nanofiltration membranes (NF) as a promising solution for water treatment. Nanofiltration membranes have more environmentally friendly operating conditions thanks to the lower energy requirements than reverse osmosis (RO) membrane systems [4,5,6,7].

One of the main drawbacks of membrane systems is membrane fouling, which can occur as particulate/colloidal fouling, organic/inorganic fouling, and biofouling [8]. Biofouling is when a biofilm develops the accumulation of bacteria and extracellular polymeric substances (EPS). Biofilm formation occurs in five stages: (i) Motile planktonic bacteria cells attach to the surface; (ii) the attachment becomes irreversible when bacteria cells aggregate to form microcolonies and start excreting EPS; (iii) cell-to-cell adhesion occurs forming multi-layered clusters, and a biofilm is formed; (iv) the biofilm matures and grows into dense mushroom-shaped structures; the EPS matrix provides protection against environmental threats; (v) the biofilm reaches a critical mass and disperses planktonic bacteria cells that will colonize other surfaces [9,10]. Biofouling causes operational system performance decline, such as pressure drop increase, flux decline, and increase in the salt passage [11]. Biofouling has been reported to contribute to more than 45% of all membrane fouling, and it has been considered a significant problem in nanofiltration and reverse osmosis membrane systems as it increases the energy demands and the overall water cost [12]. Biofouling could increase operational costs up to 30% and the overall water cost [13,14]. Biofouling has been defined as the “Achilles heel” of membrane processes [15]. Even after 99.9% of bacterial cell removal, the remaining bacteria can use the biodegradable nutrients in the feed water to develop a biofilm. Major factors contributing to biofouling are nutrient concentration in the feed water and shear forces in the system [15].

The first significant factor influencing biofilm development is the nutrient concentration in the feed water. Consequently, nutrient limitation has been proposed as a biofouling control strategy for membrane systems [16,17,18]. Recent research has focused on analyzing the effect of varying phosphorus concentrations in the feed water to control biofouling or enhance membrane cleaning strategies [19,20]. One of the challenges in applying this approach is to define the phosphorus concentration threshold at which microorganisms could inhibit their growth, as the detection limit of current techniques for measuring phosphorus in water does not go below the microgram per liter level.

**Figure 1:**
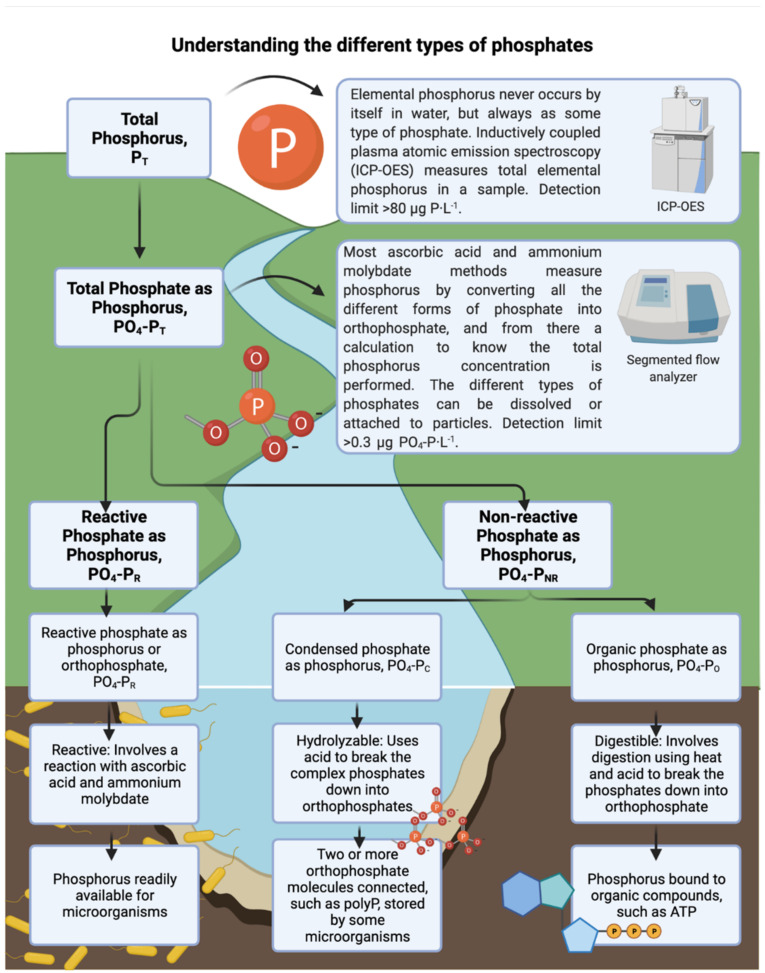
Total phosphorus and its different types of phosphates in water. Adapted from [21,29,30].

Elemental phosphorus never occurs in water but always as some type of phosphate [21]. Most of the quantification methods measure the concentration of different kinds of phosphates, and from there a calculation to obtain the elemental phosphorus concentration is performed. Phosphates can be orthophosphate (reactive form), condensed, and organic (non-reactive forms). Reactive phosphate or orthophosphate is readily available for microbial utilization [16]. Non-reactive phosphate includes condensed and organic phosphates. Condensed phosphates (like meta, pyro, and polyphosphate) are multiple orthophosphate molecules joined by an oxygen atom [22]. Organic phosphates are phosphates bound to organic compounds [23,24]. Under phosphorus limitation, bacteria can convert the less reactive forms of phosphate (condensed and organic) into orthophosphate, increasing the biodegradable phosphorus concentration in the water [25] to promote bacterial survival and growth (Figure 1).

Other significant factors influencing biofilm development are the hydrodynamics and shear forces in the flow channel. Vrouwenvelder et al. (2009) [26] demonstrated that increasing the crossflow velocity from 0.04 to 0.24 m·s^−1^ in a membrane fouling simulator increased the biomass accumulated on a reverse osmosis membrane translating into a higher pressure drop increase. As for the impact of permeation on biofilm development, previous studies have concluded that permeation does not influence biofilm development and hence has no effect on membrane performance parameters [27]. The conclusions follow the assumption that the perpendicular component of the permeation flow velocity is neglectable (for NF around 1.1 × 10^−5^ m·s^−1^ and for RO around 4.0 × 10^−6^ m·s^−1^) compared with the higher parallel component of the crossflow velocity (at least 0.1 m·s^−1^) [4,28]. Nevertheless, the effect of permeation on biofilm development in membrane systems under phosphorus limiting conditions has not been evaluated.

This study analyzed the effect of permeation and dosed phosphorus concentration (0 and 25 μg P·L^−1^), at a dosed assimilable organic carbon concentration of 250 μg C·L^−1^, on biofilm development in membrane fouling simulators (MFSs) and system performance. The MFSs operated under permeation (constant flux of 15.6 L·m^2^·h^−1^), and no permeation conditions were run for 4.7 days. Feed channel pressure drop, transmembrane pressure, and flux were used as membrane performance parameters. Optical coherence tomography (OCT) images and biomass quantification were used to analyze the different biofilms developed. We quantified the total phosphorus concentration accumulated on the membrane and spacer using microwave digestion followed by measurements with an inductively coupled plasma atomic emission spectroscopy (ICP-OES). Different studies in lab-scale have been done excluding the permeation force [19,20]. To our knowledge, this is the first time a study compares the effect of permeation and non-permeation conditions under different phosphorous concentrations on biofilm development and quantifies the phosphorus accumulated on the membrane and on the spacer to analyze the effect of phosphorus distribution on biofilm development in membrane systems.

## 2. Materials and Methods

### 2.1. Feed Water Composition

We used tap water without chlorine as the feed water for this study from the desalination plant at Thuwal, Saudi Arabia [18]. The feed water was supplemented with nutrients at a constant biodegradable carbon and nitrogen concentration of 250 μg C·L^−1^ and 25 μg N·L^−1^, respectively, and two dosed biodegradable phosphorus concentrations (0, and 25 μg P·L^−1^) (Table 1). Previous biofilm studies have been performed using tap water in this study [18,31]. Biofilm growth in the MFSs was promoted by dosing to the feed water sodium (phosphate, nitrate, and acetate) from Sigma Aldrich (Darmstadt, Germany). The dosed carbon and nitrogen concentrations were selected based on previous biofilm studies [32,33]. We used the 0 µg P·L^−1^ phosphorus dosed concentration as previous research has suggested phosphorus limitation as a biofilm mitigation strategy [16,17,34]. We selected the dosed phosphorus concentration of 25 µg P·L^−1^ following a C:N:P ratio of 100:20:10, as used in several biofouling experiments [18,27,35]. To inhibit bacterial growth in the dosed nutrient solution, the pH value was set at 11 by dosing sodium hydroxide.

### 2.2. Phosphorus Concentration in the Feed Water

Before the nutrient dosage, the tap water had a reactive phosphate as phosphorus concentration measured from orthophosphate of 0.39 µg PO_4_-P_R_·L^−1^. Duplicate water samples were processed to measure the orthophosphate in the feed water (orthophosphate in the tap water plus the orthophosphate dosed) using a segmented flow analyzer (SEAL Auto Analyser 3 HR Seal Analytical, Germany). We used the ascorbic acid and ammonium molybdate method as previously described by [23,36]. The detection limit of the segmented flow analyzer for reactive phosphorus from orthophosphate is higher than 0.30 µg PO_4_-P_R_·L^−1^. The results agree with what was dosed, and they are described in Section 3.4.

### 2.3. Experimental Setup and Operational Parameters

**Figure 2:**
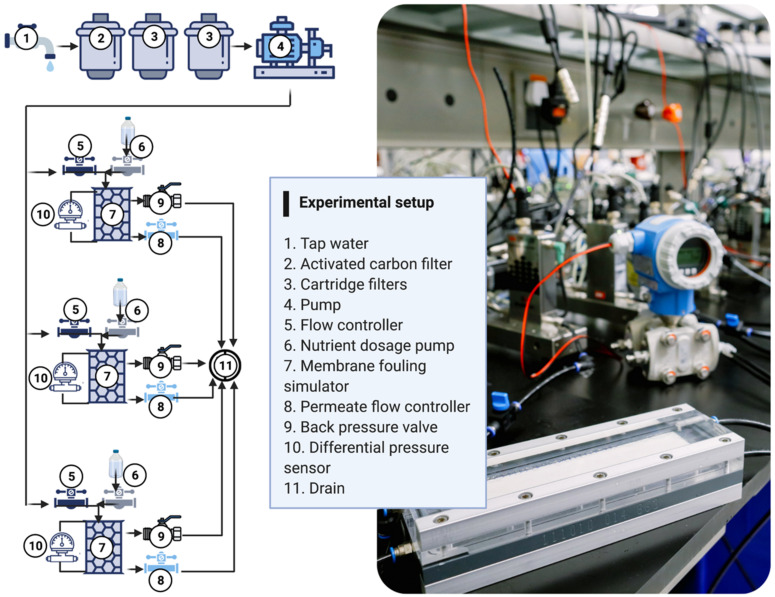
Experimental setup schematic and picture. All experiments were run in triplicate independent membrane fouling simulators for each dosed phosphorus concentration and permeation conditions.

Residual chlorine was removed by passing the tap water through an activated carbon filter as in previous studies [37]. The dechlorinated tap water then flowed through two cartridge filters (pore size 4 µm) to remove any possible particles from the activated carbon filter. The water was then pumped into the system as described in Figure 2 (Bronkhorst, Ruurlo, Netherlands). The system was composed of: (i) A flow controller, (ii) a nutrient dosage pump, (iii) a membrane fouling simulator, (iv) a permeate flow controller, (v) a back pressure valve, and (iv) a differential pressure sensor.

Twelve independent membrane fouling simulators (MFS: [38] with and without permeation were run in triplicates as described in Table 1. The membrane placed in the MFS was a polyamide thin-film composite nanofiltration (NF) membrane (NF90-400 DOW) with active permeation dimensions of 20 cm × 4 cm. A 31 mil (787 µm) thick feed channel spacer was used with dimensions of 20 cm × 4 cm and a porosity of 0.85 [39]. We wanted to differentiate the effect of permeation on biofilm development. Therefore, the horizontal feed water flow was 12.5 L·h^−1^, corresponding to a linear flow velocity of 0.13 m·s^−1^ [40], and the vertical permeate flux was set constant at 15.6 L·m^2^·h^−1^. The same MFSs were used for the no permeation conditions, but the permeate flux was set at 0 L·m^2^·h^−1^. Nutrients were dosed for 4.7 days to enhance biofilm development at a low flow rate of 0.03 L·h^−1^ to prevent the high pH nutrient solution affecting the feed water pH of 7.8 [41]. The transmembrane pressure increase and the feed channel pressure drop over the MFS were used to monitor membrane performance over time. The initial transmembrane pressure and pressure drop registered in each MFS were 1.93 ± 0.05 bar and 35 ± 5 mbar, respectively. After 4.7 days of MFS operation and once a substantial pressure drop increase and biofilm growth were observed in at least one of the nutrient conditions, a flux decline assessment was performed for all MFSs by varying the transmembrane pressure from 0.5 bar to 4 bar. The pressure drop increase chosen for the MFS simulates the biofouling condition that could be present at the inlet of the lead RO element [11,26,42]. The membrane and spacer, the hydraulics, and the operational conditions used in this study represent nanofiltration systems in practice [43].

### 2.4. Biofilm in Situ Visualization in the MFS

A spectral-domain Optical Coherence Tomography (OCT) with a 5× telecentric scan lens and a central light source wavelength of 930 nm was used to visualize biofilms in situ at the end of the experiment (Thorlabs Ganymede OCT system). Twenty-four images were taken at different randomized coordinates across the MFS. The images were taken with a refractive index of 1.33 and a frequency of 36 kHz. Images’ depth (z-direction) and length (x-direction) were 1.00 mm and 5.00 mm, respectively. The pixel size in the z-direction was set to 2.13 μm and in the x-direction to 10.00 μm. Matlab^®^ (MathWorks, Natick, MA, USA) was used as the image processing software as described in previous publications [20,44,45]. The image analysis was done as follows: (i) Automatically defining the membrane and (ii) determining the biofilm by performing automatic thresholding for pixels above 20 dB. The 20 dB threshold was defined based on measurements done on more than 200 images, as described in our previous studies [18,19,20,37]. An area of 5.00 mm × 0.61 mm from the bottom of the glass to below the spacer was taken to quantify the number of pixels in each OCT intensity interval. A higher intensity results from a more light-scattering biofilm. We proceed to calculate the biofilm thickness accumulated on the membrane (${\bar{L}}_{F}$) based on the average distance between the membrane surface and the top edge of the biofilm [44].

(1) $${\bar{L}}_{F}=\frac{1}{N}\overset{N}{\underset{i=1}{\sum }}L_{F,\;i}$$

where $L_{F,\;i}$ is the biofilm thickness [m], and N is the total number of measurements.

### 2.5. Total Cell Count, Adenosine Triphosphate, and Extracellular Polymeric Substances Quantification

After 4.7 days of running the experiment, we disassembled the membrane fouling simulators to quantify and characterize the biofilm biomass. Total bacterial cell counts (TCC) in the biofilm were measured by retrieving coupons of 4 × 2 cm of the biofouled membrane and spacer from the MFS’s inlet and outlet positions and using a flow cytometer, following the protocol reported by [46]. Adenosine triphosphate (ATP) measurements were done by retrieving membrane and feed spacer coupons of 4 × 4 cm from the MFS inlet and outlet positions, as described by [18,19,20,37] to obtain a homogenous liquid sample solution. The biofilm ATP concentrations were determined with a luminometer (Celsis Advance, Charles River Laboratories, Inc., Wilmington, MA, USA). Samples were measured in triplicates. Extracellular polymeric substances (EPS) quantification was done by retrieving membrane and feed spacer coupons of 4 × 4 cm, following the formaldehyde–NaOH method established by Liu et al. (2002) [47]. The treated samples were then placed in a microplate reader to determine the carbohydrates and proteins concentrations. The proteins concentration was determined by using a BCA protein assay kit (Thermo Scientific Inc., Portsmouth, NH, USA) according to the manufacturer’s guidelines. A Spectra A max 340pc microplate reader (Molecular Devices, San Jose, CA, USA) was used at an absorbance of 490 nm and 562 nm to determine the carbohydrate and protein concentrations in the samples, respectively.

### 2.6. Total Phosphorus on the Membrane and Spacer Characterization

At the end of the experiment, we retrieved coupons of 4 × 4 cm of the biofouled membrane and spacer from the MFS. In brief, membrane and spacers were placed independently in digestion test tubes, and we added 5 mL of 70% nitric acid to each tube. The membrane and spacer were digested using an ultraWAVE microwave digestion system (Milestone Srl, Sorisole BG, Italy). The ultraWAVE is used to digest samples in strong acids based on a single reaction chamber technology that combines microwave heating with a high-pressure reactor. We diluted the digested sample in 20 mL ultrapure water. We used a Perkin-Elmer Optima 8300 Inductively Coupled Plasma Optical Emission Spectrometry instrument (Norwalk, CT, USA) to determine the total elemental phosphorus (P) concentration (reactive and non-reactive) accumulated on the membrane and the spacer according to the protocol reported by Holden et al. (2007) [48]. Triplicate biofouled membrane and spacer samples and clean membranes and spacer samples were processed. We used a 177.434 nm wavelength in this study to determine the total phosphorus concentration accumulated on the membrane and the spacer. The detection limit of ICP-OES for elemental total phosphorus measurements is higher than 80 µg P·L^−1^.

## 3. Results

### 3.1. System Performance Parameters: Feed Channel Pressure Drop, Transmembrane Pressure, and Flux

**Figure 3:**
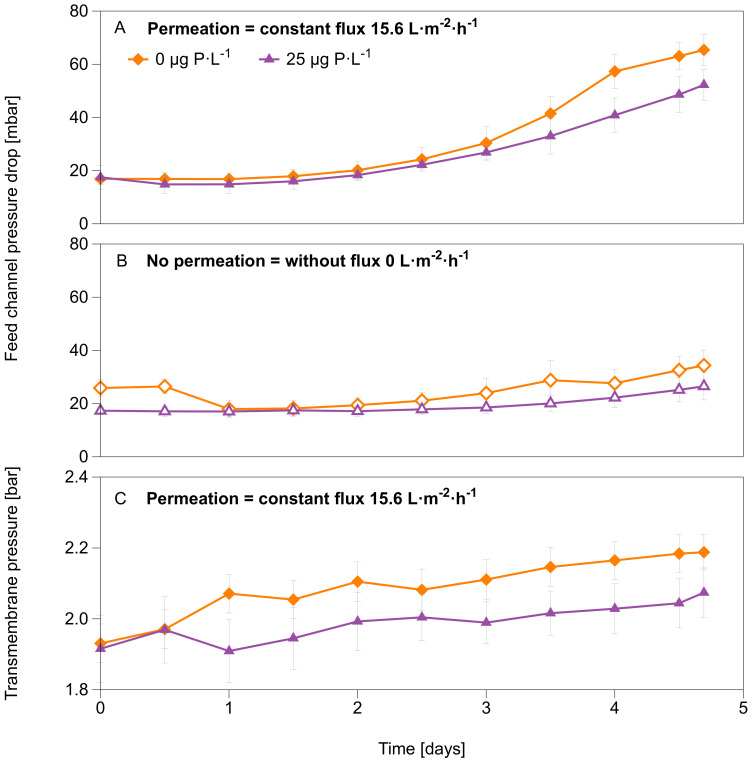
Membrane fouling simulator (MFSs) performance parameters over 4.7 days of MFSs operation. Feed channel pressure drop over time for the MFSs run under (**A**) permeation conditions at a constant flux of 15.6 L·m^2^·h^−1^, and (**B**) without permeation. (**C**) Transmembrane pressure over time for the MFSs run under permeation conditions at a constant flux of 15.6 L·m^2^·h^−1^, with varying dosed phosphorus concentrations (0 and 25 μg P·L^−1^) and a dosed assimilable organic carbon concentration of 250 μg C·L^−1^ in the feed water. The error bars represent the data of independent triplicate MFS experiments.

This study analyzed the effect of biofilms grown in MFSs with and without permeation supplied with water varying in dosed phosphorus concentrations (0 and 25 μg P·L^−1^) and a dosed assimilable organic carbon concentration of 250 μg C·L^−1^, in a nanofiltration membrane system for 4.7 days. The MFSs operated under permeation conditions were run at a constant flux of 15.6 L·m^2^·h^−1^. Figure 3A,B show that permeation had a more substantial effect on the feed channel pressure drop than no permeation for the two P concentrations tested. A higher increase in pressure drop was observed for biofilms grown at 0 compared to 25 μg P·L^−1^ condition (Figure 3A) under permeation conditions. Similarly, under no permeation conditions, on average, a higher pressure drop increase occurred for biofilms grown at 0 μg P·L^−1^ compared to biofilms grown at 25 μg P·L^−1^ (Figure 3B). At the end of the experiment, the transmembrane pressure for the MFSs operated under permeation conditions was higher (2.18 bar) for biofilms grown at 0 μg P·L^−1^ dosed phosphorus concentration than at 25 μg P·L^−1^ dosed phosphorus concentration (2.07 bar), suggesting a higher flux decline at lower phosphorus concentration conditions (Figure 3C).

**Figure 4:**
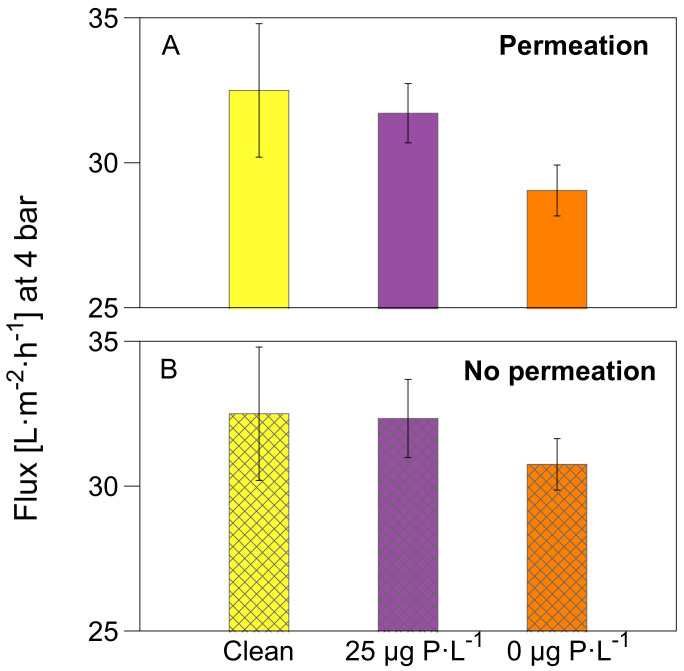
Flux at 4 bar of transmembrane pressure for the MFSs run under (**A**) permeation conditions at a constant flux of 15.6 L·m^2^·h^−1^, and (**B**) without permeation with varying dosed phosphorus concentrations (0 and 25 μg P·L^−1^) and a dosed assimilable organic carbon concentration of 250 μg C·L^−1^ in the feed water. The transmembrane pressure was varied at the end of the experiment after 4.7 days of nutrient dosage. The error bars represent the data of independent triplicate MFS experiments.

To understand how the biofilm development affected the flux at varying transmembrane pressure in all MFSs, we performed a flux decline assessment. After 4.7 days of MFS operation and biofilm growth, we varied the transmembrane pressure from 0.5 bar to 4 bar for all MFSs (run with and without permeation). The results are shown in Figure 4 for a transmembrane pressure of 4 bar and Figure S1 for transmembrane pressure from 0.5 bar to 4 bar. A higher flux decline was recorded when increasing the transmembrane pressure for MFSs run under permeation conditions compared to non-permeation conditions. Similarly, a higher flux decline is observed for MFSs run with permeation at 0 μg P·L^−1^ dosed phosphorus concentrations than 25 μg P·L^−1^ dosed phosphorus concentrations. The highest drop is observed when the transmembrane pressure is the highest (4 bar), and the phosphorus concentration is the lowest (0 μg P·L^−1^), where a flux decline of 11% is recorded compared with a clean membrane flux (Figure 4). For the MFSs operated without permeation conditions, no significant flux decline is seen for biofilms grown at 25 μg P·L^−1^; however, for biofilms grown at 0 μg P·L^−1^, a 5% flux decline is recorded compared to the clean membrane. These results suggest that the combined effect of permeation and lowering the phosphorus concentration in the feed water (0 μg P·L^−1^) had a higher impact on the membrane flux decline (11%, Figure 4A).

### 3.2. Optical Coherence Tomography Images and Biofilm Thickness on the Membrane

**Figure 5:**
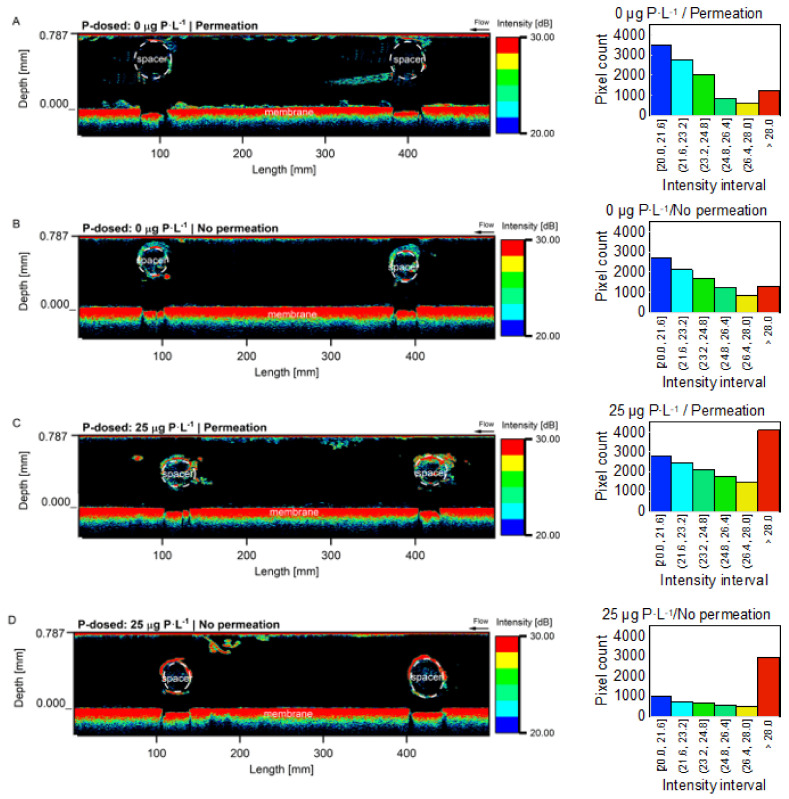
OCT images of biofilm with quantification of the intensity profile after 4.7 days of MFSs operation. Two-dimensional OCT images of the biofilms grown at (**A**,**B**) 0 μg P·L^−1^ and (**C**,**D**) 25 μg P·L^−1^ under permeation at a constant flux of 15.6 L·m^2^·h^−1^ and no permeation conditions with a dosed assimilable organic carbon concentration of 250 μg C·L^−1^ in the feed water. The OCT signal intensity was used to describe biofilm properties, with higher intensity resulting from a more light-scattering biofilm. The arrows indicate the crossflow direction.

**Figure 6:**
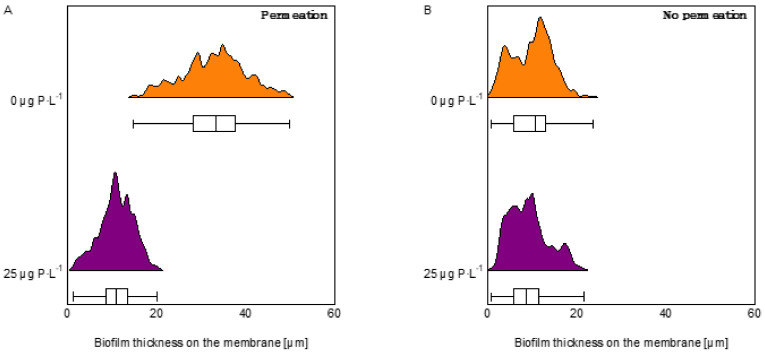
Quantification and histogram of biofilm thickness on the membrane. MFSs operated under (**A**) permeation at a constant flux of 15.6 L·m^2^·h^−1^ and (**B**) without permeation conditions at 0 and 25 μg P·L^−1^ dosed phosphorus concentrations and a dosed assimilable organic carbon concentration of 250 μg C·L^−1^ in the feed water. The graph shows the data distribution of 24 images for each phosphorus condition.

OCT images show that, in general, more biofilm accumulated in the MFSs under permeation conditions than non-permeation conditions. Under permeation conditions, the biofilms grown at 0 μg P·L^−1^ had a higher spread in the flow channel, explaining the higher effect on pressure drop increase, compared to biofilms grown at 25 μg P·L^−1^ (Figure 5). For the MFSs operated under no permeation conditions, even though there is no significant effect on pressure drop increase, the OCT images show the presence of compacted biofilm around the spacer. At a higher phosphorus concentration (25 μg P·L^−1^), the biofilm intensity around the spacer increases too. Biofilm thickness on the membrane for MFSs operated under permeation conditions was 33.3 ± 5.1 and 11.0 ± 2.4 μm for biofilms grown at 0 and 25 μg P·L^−1^, respectively (Figure 5A). For the MFSs operating without permeation, the biofilm thickness on the membrane was 10.7 ± 5.0 and 8.6 ± 2.9 μm for biofilms grown at 0 and 25 μg P·L^−1^, respectively (Figure 5B). In general, more biofilm accumulated on the membrane under permeation conditions compared to non-permeation conditions. Thicker biofilm accumulation on the membrane, under permeation conditions, was more pronounced at the low phosphorous concentration. For the MFSs run under no permeation conditions, there is no significant difference in the biofilm thickness accumulated on the membrane at different dosed phosphorus concentrations (Figure 6 and Figure S2). In summary, more biofilm accumulated on the membrane for the MFSs run under permeation conditions at a lower phosphorus concentration, translating into a higher effect on flux decline.

### 3.3. Biomass Quantification

**Figure 7:**
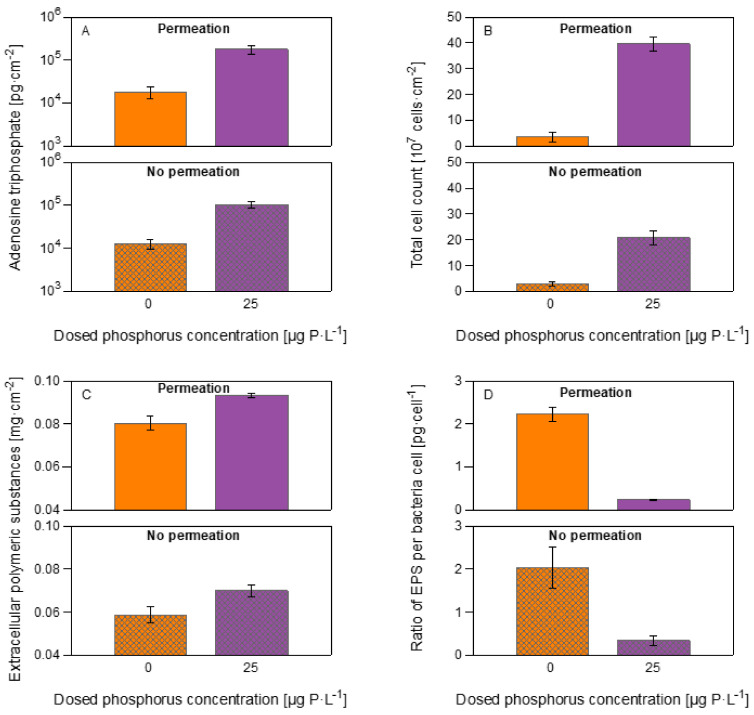
Quantification of biomass parameters after 4.7 days of MFSs operation. (**A**) Adenosine triphosphate, (**B**) total cell count, (**C**) extracellular polymeric substances (EPS) in terms of proteins and carbohydrates, and (**D**) ratio of EPS per bacteria cell of the biofilms developing on the MFSs under conditions at a constant flux of 15.6 L·m^2^·h^−1^ and no permeation conditions with varying dosed phosphorus concentrations (0 and 25 μg P·L^−1^) and a dosed assimilable organic carbon concentration of 250 μg C·L^−1^ in the feed water. The error bars represent the data of independent triplicate MFS experiments.

Figure 7A shows the adenosine triphosphate (ATP) for all the MFSs at the two phosphorous conditions. The ATP concentration for the MFSs run under permeation conditions was 17,931 ± 5414 and 178,218 ± 38,954 pg·cm^−2^ for biofilms grown at 0 and 25 μg P·L^−1^, respectively. For MFSs operated without permeation conditions, ATP was 12,569 ± 3316 and 104,717 ± 20,601 pg·cm^−2^ for biofilms grown at 0 and 25 μg P·L^−1^, respectively. There was, on average, a higher ATP concentration between MFSs operating under permeation compared without permeation conditions. As anticipated, ATP increased as phosphorus concentration increased.

The total cell count concentration is shown in Figure 7B. For the MFSs run under permeation conditions, the TCC was 3.6 ± 1.9 and 39.5 ± 4.6 × 10^7^ cells·cm^−2^ for biofilms grown at 0 and 25 μg P·L^−1^, respectively. For MFSs operated without permeation conditions, TCC was 2.8 ± 0.8 and 20.7 ± 2.6 × 10^7^ cells·cm^−2^ for biofilms grown at 0 and 25 μg P·L^−1^, respectively. Like the ATP concentration, there was on average a higher TCC concentration between MFSs operating under permeation compared without permeation conditions. TCC increased as phosphorus concentration increased.

Figure 7C shows the quantification of the extracellular polymeric substances (EPS) in terms of proteins and carbohydrates. For the MFSs run under permeation conditions the EPS was 0.080 ± 0.003 and 0.093 ± 0.001 mg·cm^−2^ for biofilms grown at 0 and 25 μg P·L^−1^, respectively. For MFSs operated without permeation conditions, EPS was 0.059 ± 0.003 and 0.070 ± 0.002 mg·cm^−2^ for biofilms grown at 0 and 25 μg P·L^−1^, respectively. EPS on MFSs operated under permeation conditions is higher compared with the MFSs run without permeation. For the MFSs run under permeation conditions, the ratio of EPS per bacteria cell was 2.22 ± 0.17 and 0.23 ± 0.02 pg·cell^−1^ for biofilms grown at 0 and 25 μg P·L^−1^, respectively. For MFSs operated without permeation conditions, the ratio of EPS per bacteria cell was 2.03 ± 0.46 and 0.34 ± 0.10 pg·cell^−1^ for biofilms grown at 0 and 25 μg P·L^−1^, respectively. The ratio of EPS per bacteria cell increases as phosphorus concentration decreases (Figure 7D).

In summary, higher biomass in terms of ATP, TCC, and EPS accumulates in the MFS operated under permeation conditions than those run without permeation. ATP and TCC decrease as phosphorus concentration decreases. The ratio of EPS production per bacteria cell increases as phosphorus concentration decreases. Therefore, biomass quantification confirmed the OCT images, where higher biomass in biofilms grown with permeation conditions translated into differences in biofilm localization affecting membrane performance indicators.

### 3.4. Phosphorus Measurements

**Figure 8:**
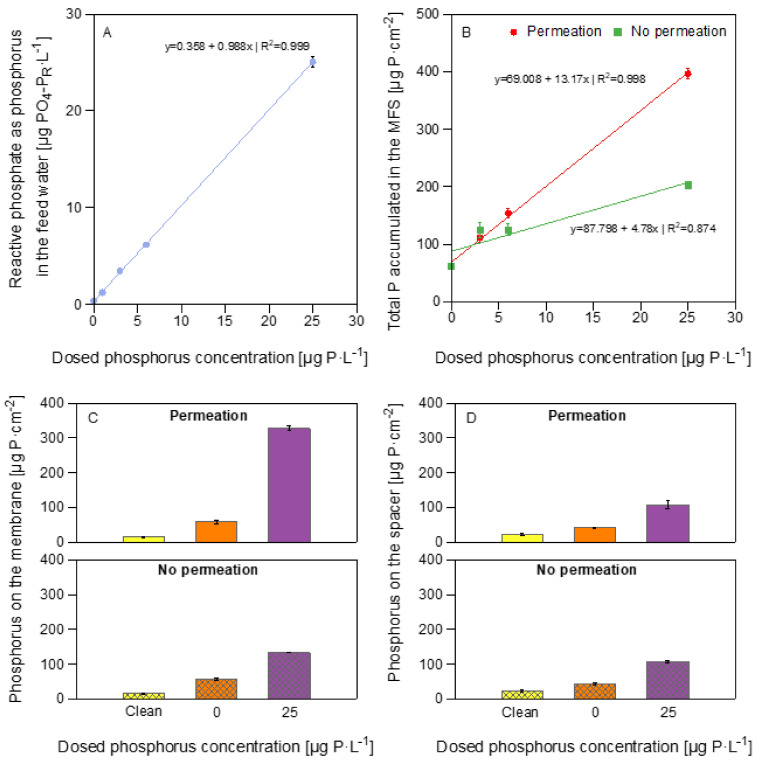
Reactive phosphorus concentration in the feed water and phosphorus concentrations on the MFSs. (**A**) XY scatter plot of the reactive phosphorus concentration in the feed water at different dosed phosphorus concentrations (0, 1, 3, 6, and 25 μg P·L^−1^) determined by a segmented flow analyzer. (**B**) XY scatter plot of the total accumulated phosphorus concentration in the MFS for permeation and non-permeation conditions determined by inductively coupled plasma atomic emission spectroscopy (ICP-OES) after 4.7 days of system operation. The dots represent the experimental data. The lines represent the fitted model. Phosphorus concentration accumulated on (**C**) the membrane, and (**D**) the spacer for the MFSs at varying dosed phosphorus concentrations (0 and 25 μg P·L^−1^) and a dosed assimilable organic carbon concentration of 250 μg C·L^−1^ in the feed water. The error bars represent the data of independent triplicate MFS experiments.

The reactive phosphate as phosphorus concentration in the feed water was quantified using a segmented flow analyzer at different dosed phosphorus concentrations (0, 1, 3, 6, and 25 μg P·L^−1^). ICP-OES could not determine the total phosphorus concentration in the feed water as it is below the device’s detection limit (>80 µg P·L^−1^). The results have an accurate correlation factor of 0.999 (Figure 8A), indicating a reactive phosphate as phosphorus concentration in the feed water, before any additional dosing, of 0.39 µg PO_4_-P_R_·L^−1^. The results of the reactive phosphate as phosphorus concentration in the feed water agree with previous studies [20].

At the end of the experiment, we extracted the phosphorus accumulated in the MFS by independently digesting the membrane and spacer. We proceeded to measure the phosphorus concentration by inductively coupled plasma atomic emission spectroscopy (ICP-OES). Figure 8B shows the relation between the dosed phosphorus concentration and the phosphorus accumulated in the MFS (membrane and spacer). MFSs run under permeation conditions showed a phosphorus accumulation on the membrane and spacer of 100.3 ± 3.5 and 435.1 ± 10.0 μg·cm^−2^ for biofilms grown at 0 and 25 μg P·L^−1^, respectively. For the MFSs run without permeation, the phosphorus accumulation on the membrane and spacer was 100.3 ± 2.9 and 240.8 ± 2.0 μg·cm^−2^ for biofilms grown at 0 and 25 μg P·L^−1^, respectively. In general, at a higher dosed phosphorus concentration, more phosphorus accumulated in the MFS run under permeation conditions compared with the ones run without permeation.

As expected, a higher phosphorus concentration accumulated on the membrane and spacer at a higher dosed phosphorus concentration. Under permeation conditions, there is a correlation between the phosphorus dosed and what accumulates on the membrane. Note that the model in Figure 8B for the MFSs run under permeation conditions has a higher correlation coefficient (R^2^ = 0.998) than the no permeation conditions (R^2^ = 0.874). Figure 8C,D show separately the phosphorus concentration accumulated on the membrane and the spacer, respectively. From the total phosphorus accumulated in the MFS, more phosphorus accumulates on the membrane compared to the spacer, regardless of the permeation condition. Therefore, permeation impacted the phosphorus accumulated on the membrane, and no effect is observed on the phosphorus accumulation on the spacer. A higher phosphorus accumulation on the membrane was observed for biofilms grown under permeation conditions than those run without permeation. These findings show that phosphorus distributes differently on the MFS depending on the dosed phosphorus concentration and permeation conditions, which causes the differences in the biofilm localization, and, therefore, the effect on the membrane performance parameters.

Table 2 shows the phosphorus amount after 4.7 days of MFS operation. For biofilms grown at 25 μg P·L^−1^, 90% of what was in the feed water (P in tap and P dosed) accumulated in the MFS under permeation conditions and 46% without permeation conditions. On the contrary, for biofilms grown at 0 μg P·L^−1,^ more phosphorus accumulated in the MFS than what was dosed and present as orthophosphate in the water. These findings suggest that under limited phosphorus conditions, bacteria use non-reactive sources of phosphorus that should be present in the feed water but that are challenging to measure for biofilm development and growth.

## 4. Discussion

### 4.1. Permeation Caused a Faster Decline in System Performance

The shear forces and hydrodynamics in the flow channel influence biofilm development [15]. In crossflow membrane systems, there are two simultaneous flow velocities: (i) The feed crossflow velocity, flowing parallel to the membrane walls, and (ii) the permeate crossflow velocity, flowing perpendicular to the membrane surface [49]. Previous studies performed in nanofiltration membranes in a monitor, test rigs, a pilot-scale, and a full-scale installation demonstrated that, irrespective of whether a flux was applied or not, the feed channel pressure drop and ATP increased [27]. Some of the assumptions to discard permeation in membrane studies are based on the low perpendicular component of the permeation flow velocity (for NF around 1.1 × 10^−5^ m·s^−1^ and for RO around 4 × 10^−6^ m·s^−1^) compared with the higher parallel component of the crossflow velocity (between 0.10 to 0.40 m·s^−1^) [4,28]. The literature conclusions regarding the importance of considering permeation in membrane studies are therefore contradictory. In ultrafiltration membranes, Eshed et al. (2008) [49] found the impact of the permeate drag force on the biofouling layer very important and concluded that the permeate flow enhanced biofilm development. While our study used NF membranes with lower permeate flux, the results confirm similarly that permeation had an impact on biofilm development. In this study, a higher pressure drop increase and higher flux decline were observed for biofilms grown under permeation conditions than non-permeation conditions. Overall, more biomass with higher EPS developed in MFS with permeation compared without permeation at the two dosed phosphorus concentration conditions (Figure 7C). Flux induces convective transport of nutrients and solutes to the membrane surface [50,51]; therefore, the impact of flux on biofilm development varied depending on the phosphorous concentration.

### 4.2. Permeation Impact on Biofilm Development Varied Depending on the Nutrient Condition

The effect of permeation on biofilm localization varied in extent depending on the phosphorous concentration tested. Previous studies showed that, in the presence of a feed spacer, biofilm starts to develop first at the spacer [52,53]. When no nutrient limitations existed in the 25 μg P·L^−1^ condition, biofilm started to develop and expand on the spacer, and permeation resulted in more biomass without a major shift in biofilm localization. On the other hand, at the 0 μg P·L^−1^, the permeation effect on biofilm localization was more pronounced. More biofilm coverage was observed on the membrane under permeation than no permeation conditions. Biofilms spread more to enhance nutrient capture under limiting conditions [18], and permeation force contributed to more accumulation of substrate in the membrane area resulting in a higher membrane biofilm coverage. The impact of biofilm development on performance is dependent on the area the biofilm occupies in the flow channel and biofilm characteristics such as EPS nature, concentration, and properties (EPS to bacterial cell ratio). The results from this study reaffirm that at a lower phosphorus concentration, few bacteria cells start producing more EPS [19,20]. Values of EPS to bacteria cells for different types of biofilms reported in the literature range from 0.2–4.5 [54,55]. According to a model proposed by Jin and Marshall (2020) [56], low EPS to bacterial cell ratios form compact and denser biofilms, compared to higher EPS to bacteria cell ratios where a less dense and disperse biofilm is formed with a tendency to break up. This study shows that permeation accelerated the pressure drop increase for biofilms grown at 0 more than 25 μg P·L^−1^. For the 0 μg P·L^−1^ under permeation conditions, the higher effect on pressure drop increase is explained by a higher EPS ratio per bacterial cell, which translated into “expanded” EPS in the flow channel (Figure 5) following Javier et al. (2020) [20] results. Previous studies [37] found that the dominant bacterial families for biofilms grown under 0 μg P·L^−1^ were *Sphingomonadaceae*, which are related to extracellular polymeric substances production [57,58,59]. This study’s results agree with Jin and Marshall’s model [56], where OCT images confirmed that a compact biofilm formed around the spacer (Figure 5) at higher dosed phosphorus concentration (low EPS to bacterial cell ratio). A uniform observation that permeation affects biofilm development can be made, however, with varying extent depending on the nutrient availability.

### 4.3. Practical Implications and Future Research

Since the realization of the biofouling problem in membrane systems, all efforts have been put to control biofouling. Permeation is one of the main operational parameters in the system that was investigated to control biofouling. The critical flux concept, where below certain flux biofouling would not occur, has been supported and opposed in previous studies [20,44,45]. Results from this study reinforce that with only controlling flux, biofouling cannot be controlled. This study showed that even at ultra-trace reactive phosphate as phosphorus concentration in the feed water, and no permeation, bacteria developed a biofilm, with few bacterial cells but high EPS per cell. Furthermore, results from this study highlighted that with flux conditions, biofouling could be more severe depending on nutrient availability, emphasizing the need to include flux in biofouling studies.

Further research is needed regarding feed water nutrient manipulation for biofouling control with a focus on engineering biofilms that are controllable and with enhanced cleanability through more environmentally friendly methods. At a certain threshold of low phosphorus content in the feed water, phosphorus limitation shows a promising approach to developing biofilms that are easier to control and clean with more sustainable methods. Up to today, most biofouling studies quantify the total cell count. However, little research has been done to understand the types and concentration of macromolecules inside bacterial cells, like polyphosphates, to determine the relationship between biofilm development and membrane performance parameters. Flow cytometry has proven a promising technique to characterize phosphate accumulating organisms and has been used as a polyphosphate detector [52]. These macromolecules either inside the bacterial cells or in the EPS might influence biofilm development and biofilm localization in the flow channel. Therefore, research should continue analyzing the relationship between nutrients in the feed water, biofilm localization, and the effect on membrane performance decline.

## 5. Conclusions

This study analyzed the effect of permeation on biofilm development in MFSs supplied with water varying in dosed phosphorus concentrations (0 and 25 μg P·L^−1^) and a dosed assimilable organic carbon concentration of 250 μg C·L^−1^ in a nanofiltration membrane system. The conclusions of the study can be summarized by:

(i). Permeation resulted in a faster decline in system performance (faster feed channel pressure drop increase and higher transmembrane pressure increase).
(ii). Permeation impact on biofilm development varied depending on nutrient condition with a stronger impact at low phosphorous concentration:
  ○ For the 0 μg P·L^−1^ under permeation conditions, the pressure drop increase is explained by “expanded” EPS in the flow channel, thus biofilm localization. The higher flux decline was explained by a thicker biofilm, resulting from a higher phosphorus accumulation on the membrane.
  ○ For the 25 μg P·L^−1^ under permeation conditions, the pressure drop increase is explained by a higher quantity of “condensed” EPS around the spacer, thus biofilm structure and composition.

## Figures and Tables

**Table 1:** Experimental nutrient and operational conditions for the study (experiments were run in triplicate MFSs).

Dosed C Concentration (µg C·L^−1^)	Dosed N Concentration (µg N·L^−1^)	Dosed P Concentration (µg P·L^−1^)	Permeation
250	50	0	Yes
250	50	0	No
250	50	25	Yes
250	50	25	No

**Table 2:** Phosphorus amount after 4.7 days of MFS operation.

Dosed Phosphorus Concentration	0 µg P·L^−1^	25 µg P·L^−1^
**Phosphorus IN**		
Reactive phosphate as phosphorus in the feed water, *P_R-FW_* [µg PO_4_-P_R_]	559	35,287
Reactive phosphate as phosphorus in the tap water *P_R-TAP_* [µg PO_4_-P_R_]	559	559
Reactive phosphate as phosphorus dosed, *P_R-DSD_* [µg PO_4_-P_R_]	0	34,728
**Phosphorus in the MFS under permeation conditions**		
Total phosphorus (reactive and non-reactive) accumulated on the membrane and spacer, *P_MFS_* [µg P]	4943	31,716
**Phosphorus in the MFS under no permeation conditions**		
Total phosphorus (reactive and non-reactive) accumulated on the membrane and spacer, *P_MFS_* [µg P]	4946	16,204

## Data Availability

The data that support the findings of this study are available from the corresponding author.

## Supplementary Materials

The following supporting information can be downloaded at: https://www.mdpi.com/article/10.3390/membranes12030335/s1, Figure S1: Flux at different transmembrane pressures for the MFSs run under (A) permeation conditions at a constant flux of 15.6 L·m^2^·h^−1^, and (B) without permeation with varying dosed phosphorus concentrations (0, 3, 6 and 25 μg P·L^−1^) and a dosed assimilable organic carbon concentration of 250 μg C·L^−1^ in the feed water. The transmembrane pressure was varied at the end of the experiment after 4.7 days of nutrient dosage. The graphs show the average of independent triplicate experiments; Figure S2: Quantification of biofilm thickness on the membrane after 4.7 days of MFSs operation. Histogram of biofilm thickness on the membrane for MFSs operated under (A) permeation and (B) without permeation conditions at varying dosed phosphorus concentrations in the feed water. The graph shows the data distribution of 24 images for each phosphorus condition.
