# Supplementary material for: Permeation Increases Biofilm Development in Nanofiltration Membranes Operated with Varying Feed Water Phosphorous Concentrations
Source: Membranes (Basel). 2022 Mar 18;12(3):335. doi: 10.3390/membranes12030335 (PMC8950030; doi:10.3390/membranes12030335)
Supplement: Supplementary file 1 [file membranes-12-00335-s001.zip › membranes-1610034-supplementary.pdf]

Supplementary material

Permeation influences biofilm development in membrane systems operated with varying feed water phosphorous concentrations

Luisa Javier[a], Laura Pulido-Beltran[a], Johannes S. Vrouwenvelder[a,b], and Nadia M. Farhat[a]*

[a] King Abdullah University of Science and Technology (KAUST), Water Desalination and Reuse Center (WDRC), Division of Biological and Environmental Science and Engineering (BESE), Thuwal 23955-6900, Saudi Arabia.
[b] Delft University of Technology, Faculty of Applied Sciences, Department of Biotechnology, Van der Maasweg 9, 2629 HZ Delft, The Netherlands.

* Corresponding author, e-mail: nadia.farhat@kaust.edu.sa, King Abdullah University of Science and Technology (KAUST), Water Desalination and Reuse Center (WDRC), Division of Biological and Environmental Science and Engineering (BESE), Thuwal 23955-6900, Saudi Arabia.

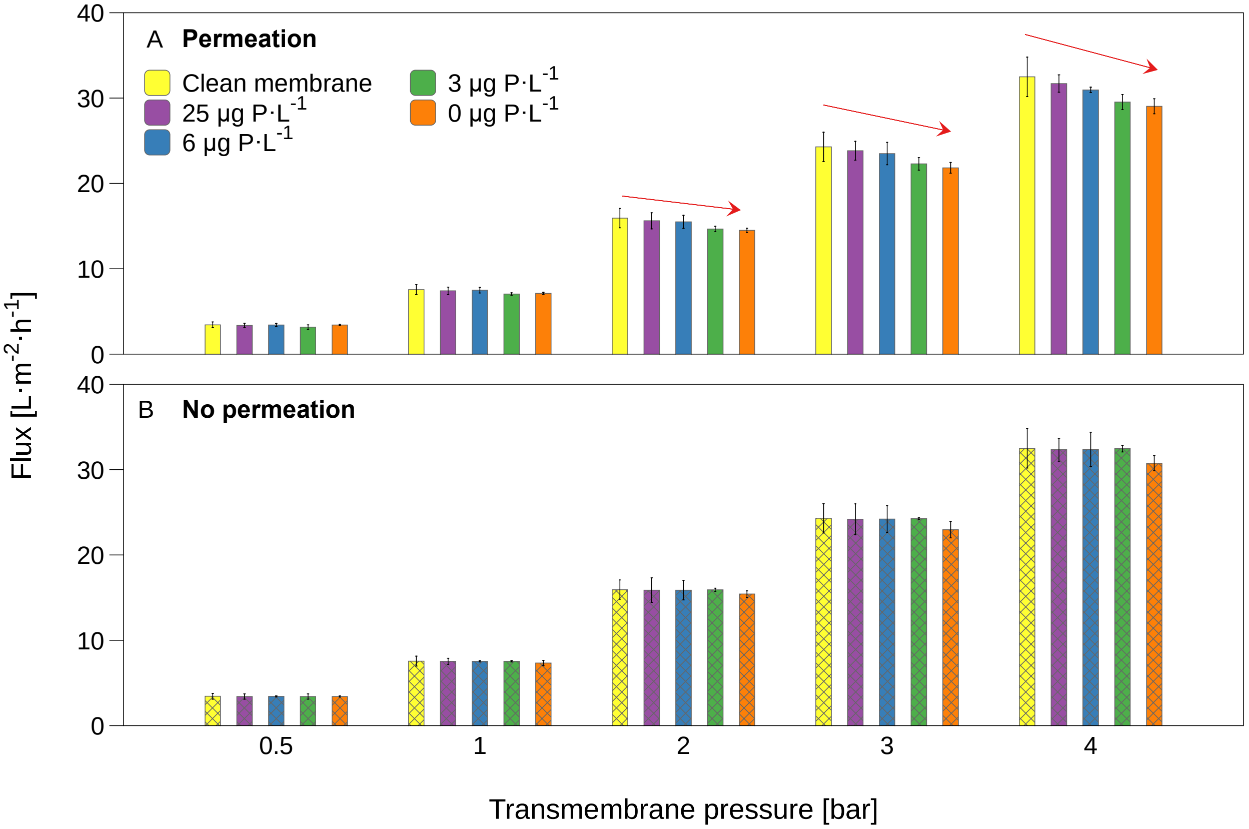

Figure S1 – Flux at different transmembrane pressures for the MFSs run under (A) permeation conditions at a constant flux of 15.6 L·m^2·h^{-1}, and (B) without permeation with varying dosed phosphorus concentrations (0, 3, 6 and 25 µg P·L^{-1}) and a dosed assimilable organic carbon concentration of 250 µg C·L^{-1} in the feed water. The transmembrane pressure was varied at the end of the experiment after 4.7 days of nutrient dosage. The graphs show the average of independent triplicate experiments.

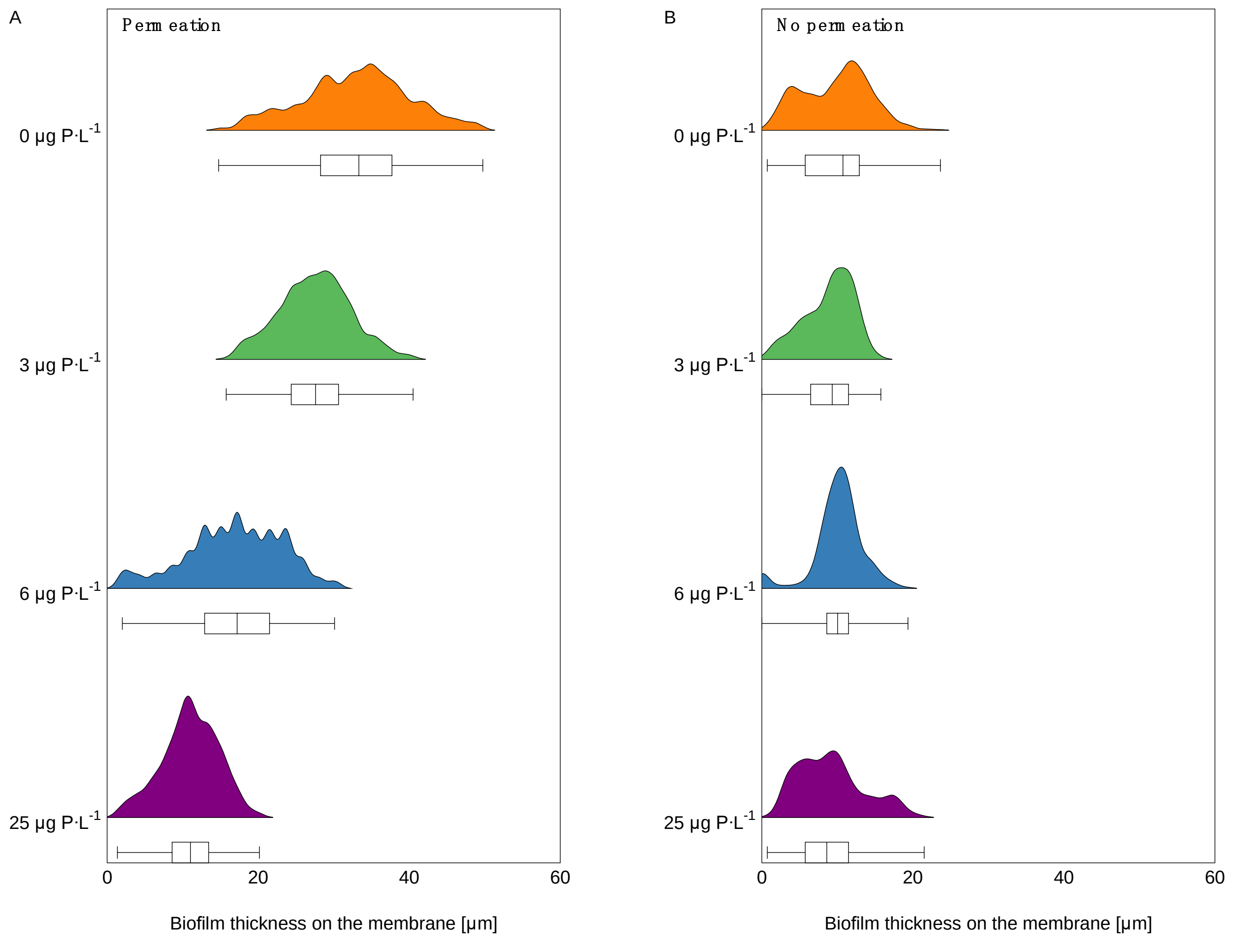

Figure S2 – Quantification of biofilm thickness on the membrane after 4.7 days of MFSs operation. Histogram of biofilm thickness on the membrane for MFSs operated under (A) permeation and (B) without permeation conditions at varying dosed phosphorus concentrations in the feed water. The graph shows the data distribution of 24 images for each phosphorus condition.
